# Gender- and region-specific changes in estrogen signaling in aging rat brain mitochondria

**DOI:** 10.18632/aging.101538

**Published:** 2018-08-31

**Authors:** Christopher M. Evola, Tanner L. Hudson, Luping Huang, Adrian M. Corbett, Debra A. Mayes

**Affiliations:** 1 Department of Neuroscience, Cell Biology and Physiology, Wright State University, Boonshoft School of Medicine, College of Science and Math, Dayton, OH 45435, USA

**Keywords:** aging, gender, ER-beta, neurodegeneration, mitochondria

## Abstract

Recently epidemiological studies suggest females lose neuroprotection from neurodegenerative diseases as they go through menopause. It has been hypothesized that this neuroprotection is hormone-dependent. The current study characterized cell signaling molecules downstream of estrogen receptor beta that are known to play a role in memory, PKC, ERK, and connexin-43, in regions of the brain associated with memory decline in an attempt to elucidate significant changes that occur post-estrus. Total whole cell lysates were compared to isolated mitochondrial protein because mitochondrial function is known to be altered during aging. As hypothesized, protein concentrations differed depending on age, gender, and brain region. Additionally, many of these changes occurred within mitochondria but not within whole cell lysates indicating that these are epigenetic alterations. These findings accentuate the complexity of aging and provide insight into the gender-specific cellular processes that occur throughout this process.

## Introduction

As people age, the prevalence of a variety of health-related symptoms commonly increase, such as: joint pain, decreased muscle mass, fatigue, anxiety and depression, and memory loss. The propensity for and the disease severity for neurodegenerative diseases such as Parkinson’s Disease, Alzheimer’s Disease, and stroke also increase with age [1–5]. Mechanisms for this age-associated phenomenon are currently unknown; however, females experience higher levels of neuroprotection than males until they reach menopause [6,7]. Post-menopause, this protection from neurologic disease diminishes resulting in lower protection than their male counterparts. This loss of protection may be one mechanism in which females have a higher incidence and severity of stroke and neurodegenerative diseases [7,8]. Because females experience a sudden drop in estrogen levels during menopause [9], it has been hypothesized that hormones may play a role in the neuroprotection noted early in female life. Furthermore, we propose that gender is an important factor to consider when evaluating the aging process.

Hormones such as estrogen and progesterone are tissue-specific signaling molecules that can regulate the activity of the cerebral cortex, hippocampus, and amygdala [10,11]. There are two isoforms of these receptors, ER-alpha and ER-beta, both of which regulate transcription [12]. Estrogen receptor beta is expressed differentially throughout the brain and has been implicated to have neuroprotective and tumor suppressing functions in the aging brain [11–17]. Estrogen receptor-beta also signals within the mitochondria. This is relevant because mitochondrial alterations have been correlated to age- and region-associated changes in learning and memory [7]; however, the mechanisms for these changes for both age and gender are unknown. Therefore, signaling cascades that are both down-stream of estrogen receptor beta and which regulate respiration within the mitochondria (PKC, cx43, and ERK) were quantified in brain regions that correspond to these mental tasks in order to begin to unravel this question.

Signaling pathways downstream of the estrogen receptor-beta including protein kinase C (PKC), extracellular signal-related kinase (ERK), and connexin were investigated in the current study. These proteins signal within the mitochondria and have been implicated to have altered activity levels throughout the aging process [14,15,18–25]. We hypothesize that changes in these estrogen-responsive proteins will be gender and brain region specific; providing insight into key signaling alterations that occur during the aging process.

In this study, we characterized age-specific and gender-specific changes that occur in five brain regions (cerebral cortex, hippocampus, amygdala, corpus callosum, and cerebellum). These regions were selected due to their known role in cognition, learning, and memory throughout the aging process. Although all of these regions are involved in cognition and memory, which may decline with age; hormones have been shown to play an important role in the regulation of the cerebral cortex, hippocampus, and amygdala [11]. Because brain regions respond to aging differentially [26], characterizing multiple regions provides a more complete description of changes that occur during the aging process, particularly as these regions are associated with different neurodegenerative diseases. This has been described in studies that have characterized gross structural, region-specific changes with age utilizing magnetic resonance imaging (MRI) and computed tomography (CT) scans. For example, the cerebral cortex, hippocampus, and cerebellum have been shown to significantly decrease in volume with age, while the amygdala undergoes very minimal anatomical change throughout the aging process [26–28]. Although large-scale morphological changes have previously been shown to occur in several of the brain regions investigated, continued exploration into what alterations occur post-menopause may be vital to our understanding of the role of estrogen depletion in the loss of neuroprotection. The aim of the current study was to characterize age-specific, gender-specific, and brain region-specific alterations in proteins downstream of the estrogen receptor. This study provides a foundation for further research, as well as, insight into the complexity of the aging process.

## RESULTS

Because neurodegenerative changes associated with Alzheimer’s disease have shown correlations with gender-dependent severity, brain regions were selected dependent upon their association with learning and memory. The cortex, hippocampus, cerebellum, and cortical white matter (corpus callosum), are all areas that have shown significant changes with age in human MRI studies. These areas are also known to be highly susceptible in Alzheimer’s disease, one example of a disease in which the susceptibility and disease severity significantly increase post-menopause in females [7].

### Cerebral cortex – whole cell

**Figure 1:**
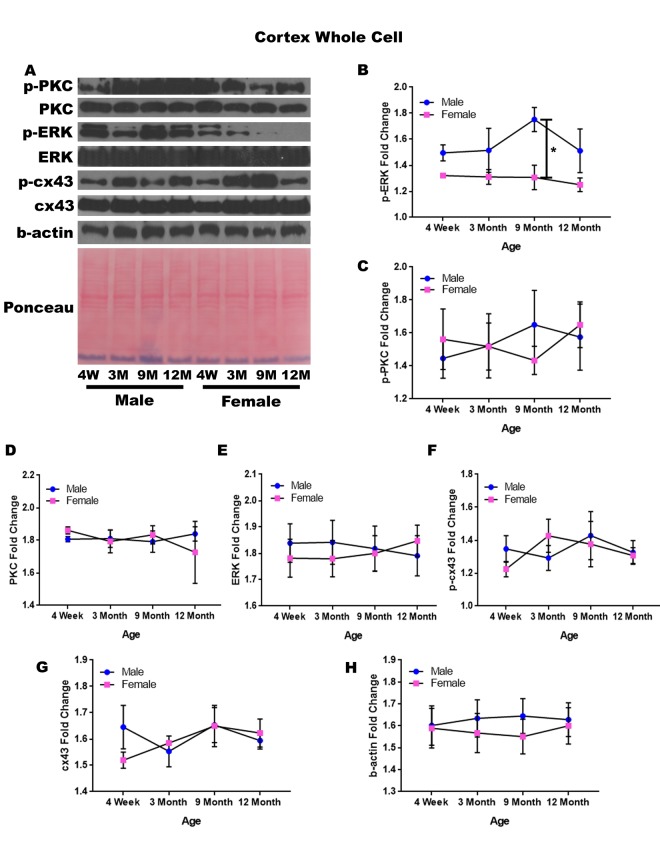
**Signaling downstream of estrogen in the cerebral cortex across age and gender.** Representative western blots for each protein of interest and a representative Ponceau stain as a load control (**A**). Graphical depiction of the fold change for p-ERK (**B**), p-PKC (**C**), ERK (**D**), ERK (**E**), p-cx43 (**F**), cx43 (**G**), beta-actin (**H**). Error bars = SEM. 4W = 4 weeks of age; 3M = 3 months of age; 9M = 9 months of age; 12M = 12 months of age. Pink = female; blue = male. ANOVA with Tukey posthoc, * = P<0.05.

In order to determine whether signaling downstream of the estrogen receptor changed within the cortex over time and between genders, protein was isolated from the cerebral cortex of male and female rats at 4 timepoints throughout their lifespan (4 weeks, 3 months, 9 months, and 12 months of age; n = 3-4/gender/age). These timepoints in the rat lifespan correspond to youth to middle-age in humans (see Supplementary Table 1). The cerebral cortex was not subdivided into specific regions, and therefore contained the entire cortex for each brain. Whole cell analysis for this region showed that the protein concentration of p-ERK was significantly different amongst the genders at nine months of age (Figure 1A, 1B; p = 0.008). The male rats had significantly higher concentrations of p-ERK compared to the female counterparts (Figure 1A, 1B; p = 0.008). Additionally, there was a trend for the twelve-month males to also have higher concentrations of p-ERK when compared to age-matched females (Figure 1A, 1B; p = 0.091). Although this was not statistically significant there was a trend which may become significant if sample numbers were increased. The concentrations of p-PKC, PKC, ERK, p-cx43, and cx43 remained relatively constant regardless of gender or age (Figure 1A, 1C-G). A graphical depiction of this data can be seen in Figure 1.

### Cerebral cortex – mitochondrial isolation

**Figure 2:**
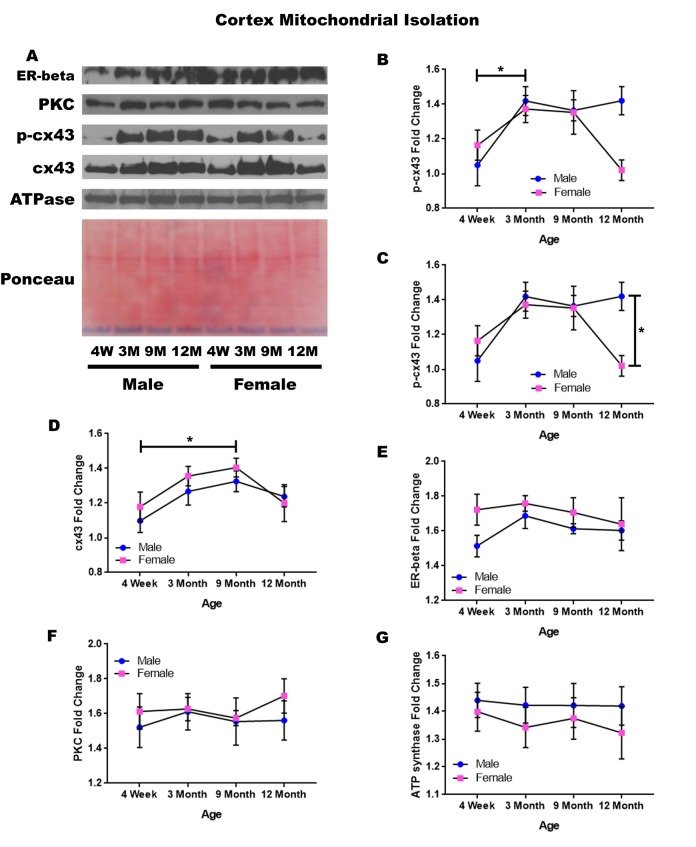
**Signaling downstream of estrogen in the mitochondria of the cerebral cortex across age and gender**. Representative western blots for each protein of interest and a representative Ponceau stain as a load control (**A**). Graphical depiction of the fold change for p-cx43 comparing age (**B**), p-cx43 comparing gender (**C**), cx43 (**D**), estrogen receptor beta (**E**), PKC (**F**), ATP synthase (**G**). Error bars = SEM. 4W = 4 weeks of age; 3M = 3 months of age; 9M = 9 months of age; 12M = 12 months of age. Pink = female; blue = male. ANOVA with Tukey posthoc, * = P<0.05.

In order to determine whether signaling downstream of the estrogen receptor changed within cortical mitochondria over time and between genders, mitochondria were isolated from the cerebral cortex of male and female rats at 4 timepoints throughout their lifespan (4 weeks, 3 months, 9 months, and 12 months of age; n = 3/gender/age). The concentration of p-cx43 significantly increased between four weeks of age and three months of age (Figure 2A & 2B; p = 0.028). There was also a significant difference between male and female rats at twelve months of age (Figure 2A & 2C; p = 0.014), with the female rats having significantly lower concentrations. Both genders experienced an increase in mitochondrial p-cx43 concentration as they aged from 4 weeks to 3 months of age (Figure 2A & 2D; p = 0.028); however, at the post-estrus age between 9 months and 12 months, females experience a decrease resulting in significantly lower p-cx43 concentration compared to their male counterparts (Figure 2A & 2C; p = 0.014).

Isolated mitochondria from the cortex also showed cx43 was found to significantly change with age (Figure 2A & 2F). Both genders experienced a significant increase in cx43 concentration between four weeks and nine months of age (Figure 2A & 2D; p = 0.021). This increase may be related to normal growth and establishment of neural circuitry which continues into young adulthood.

To start to identify the role of estrogen in mitochondrial signaling, we analyzed the concentration of the estrogen receptor beta, which showed a strong trend for gender differences at four weeks of age. The four-week-old female rats had a strong trend showing elevated concentrations of estrogen receptor beta when compared to the four-week-old male rats (Figure 2A & 2D; p = 0.056).

The concentration of PKC remained relatively constant when comparing both age and gender in mitochondria isolated from the cortex (Figure 2A & 2C). A graphical depiction of this data and the alterations demonstrated can be visualized in Figure 2.

### Hippocampus – whole cell

**Figure 3:**
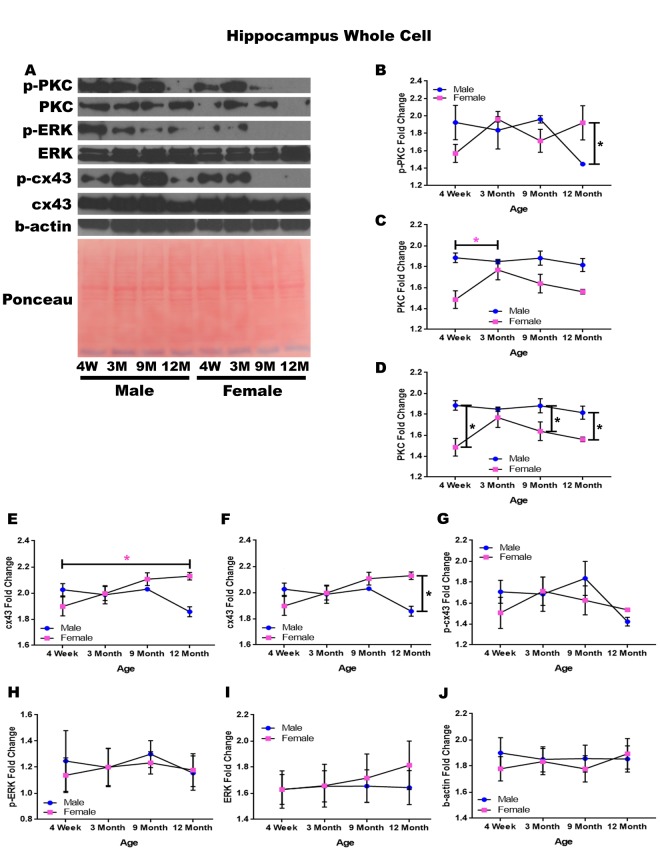
**Signaling downstream of estrogen in the hippocampus across age and gender**. Representative western blots for each protein of interest and a representative Ponceau stain as a load control (**A**). Graphical depiction of the fold change for p-PKC (**B**), PKC comparing age (**C**), PKC comparing gender (**D**), cx43 comparing age (**E**), cx43 comparing gender (**F**), p-cx43 (**G**), p-ERK (**H**), ERK (**I**), beta-actin (**J**). Error bars = SEM. 4W = 4 weeks of age; 3M = 3 months of age; 9M = 9 months of age; 12M = 12 months of age. Pink = female; blue = male. ANOVA with Tukey posthoc, * = P<0.05. Pink * = p<0.05 for females only.

In order to determine whether signaling downstream of the estrogen receptor changed within the hippocampus over time and between genders, whole cell lysate proteins were isolated from the hippocampus of male and female rats at 4 timepoints throughout their lifespan (4 weeks, 3 months, 9 months, and 12 months of age; n = 3-4/gender/age). Significant differences in p-PKC were found when comparing males and females at twelve months of age with males experiencing significantly lower concentrations than their female counterparts (Figure 3A & 3B; p = 0.028). Although not statistically significant, there was also a trend implicating gender differences at four weeks of age (Figure 3A & 3B; p = 0.090), and a decrease in p-PKC in males between nine months and twelve months of age (Figure 3A & 3B; p = 0.079). Larger sample sizes may elucidate these findings.

Hippocampal whole cell analysis also demonstrated a significant increase of PKC concentration within female rats between four weeks of age and three months of age (Figure 3A & 3C; p = 0.037). In addition to age-dependent alterations, there were also significant PKC concentration differences between genders at four weeks of age (Figure 3A & 3D; p <0.001), nine months of age (Figure 3A & 3D; p = 0.020), and twelve months of age (Figure 3A & 3D; p = 0.015) with males experiencing higher concentrations at all time points.

Both gender and age-dependent changes were noted for cx43 (Figure 3A, 3E, & 3F). Female rats experienced a significant increase in cx43 from four weeks of age to twelve months of age (Figure 3A & 3E; p = 0.022), and female rats also demonstrated significantly higher concentrations of cx43 at twelve months of age compared to their male counterparts (Figure 3A & 3F; p = 0.002). There were no significant alterations noted within p-cx43, p-ERK, or ERK concentrations across gender or age (Figure 3A, 3G-I). A graphical depiction of this data can be seen in Figure 3.

### Hippocampus - mitochondrial isolation

**Figure 4:**
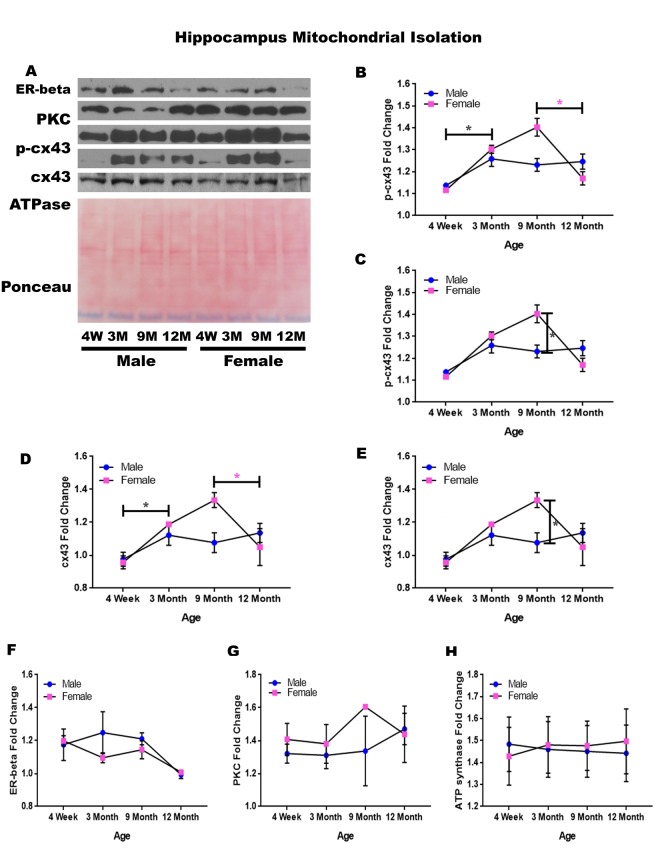
**Signaling downstream of estrogen in the mitochondria of the hippocampus across age and gender**.Representative western blots for each protein of interest and a representative Ponceau stain as a load control (**A**). Graphical depiction of the fold change for p-cx43 comparing age (**B**), p-cx43 comparing gender (**C**), cx43 comparing age (**D**), cx43 comparing gender (**E**), estrogen receptor beta (**F**), PKC (**G**), ATP synthase (**H**). Error bars = SEM. 4W = 4 weeks of age; 3M = 3 months of age; 9M = 9 months of age; 12M = 12 months of age. Pink = female; blue = male. ANOVA with Tukey posthoc, * = P<0.05. Pink * = p<0.05 for females only.

In order to determine whether signaling downstream of the estrogen receptor changed within hippocampal mitochondria over time and between genders, mitochondria were isolated from the hippocampus of male and female rats at 4 timepoints throughout their lifespan (4 weeks, 3 months, 9 months, and 12 months of age; n = 3-4/gender/age). A significant increase in p-cx43 was found for both genders from four weeks of age to three months of age (Figure 4A & 4B; male p = 0.031, female p = 0.001). This increase was then followed by a significant decrease of p-cx43 from nine months of age to twelve months of age (Figure 4A & 4B; p = 0.009). Additionally, female rats possessed a significantly higher concentration of p-cx43 at nine months of age compared to their male counterparts (Figure 4A & 4C; p < 0.001).

Isolated mitochondria from the hippocampus also showed altered expression of cx43. There was a significant increase in cx43 from four weeks of age to three months of age (Figure 4A & 4D; p = 0.012). Similar to the alterations found with p-cx43, there was a significant decrease in mitochondrial cx43 at twelve months of age (Figure 4A & 4D; p = 0.017); however, this was only seen in females. There was a significant difference between male and female cx43 expression at nine months of age, with female rats having significantly more cx43 when compared to their male counterparts (Figure 4A & 4E; p = 0.003).

Although not significantly different, the estrogen receptor beta displayed a decreasing trend between nine and twelve months of age (Figure 4A & 4B; p = 0.089). The concentration of PKC remained relatively constant regardless of age or gender (Figure 4A & 4G). A graphical depiction of this data is presented in Figure 4.

### Amygdala – whole cell

**Figure 5:**
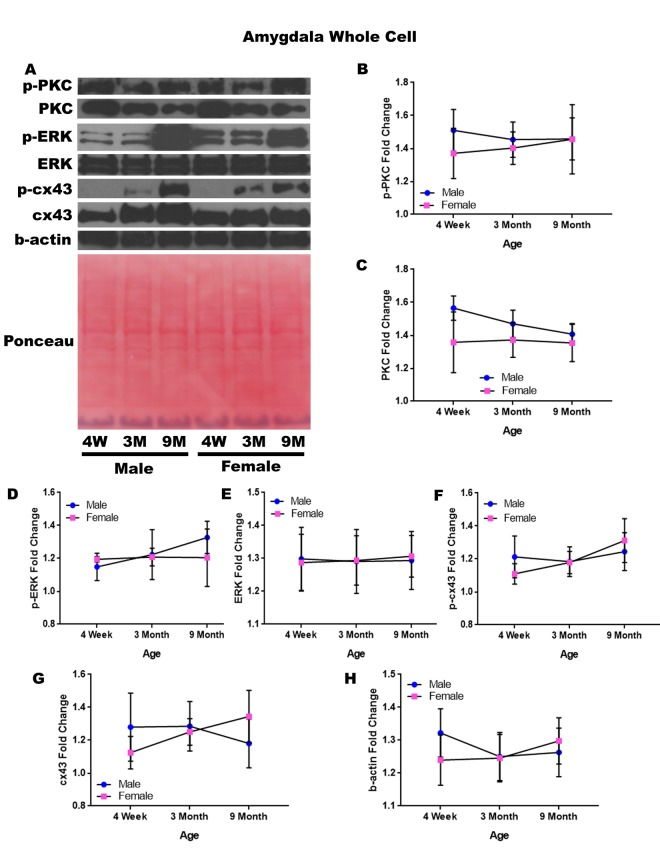
**Signaling downstream of estrogen in the amygdala across age and gender**. Representative western blots for each protein of interest and a representative Ponceau stain as a load control (**A**). Graphical depiction of the fold change for p-PKC (**B**), PKC (**C**), p-ERK (**D**), ERK (**E**), p-cx43 (**F**), cx43 (**G**), beta-actin (**H**). Error bars = SEM. 4W = 4 weeks of age; 3M = 3 months of age; 9M = 9 months of age; 12M = 12 months of age. Pink = female; blue = male. ANOVA with Tukey posthoc, * = P<0.05.

In order to determine whether signaling downstream of the estrogen receptor changed within the amygdala over time and between genders, whole cell lysate proteins were isolated from the amygdala of male and female rats at 3 timepoints throughout their lifespan (4 weeks, 3 months, and 9 months of age; n = 3/gender/age). No animals at the 12-month timepoint were analyzed for this brain region (Figure 5A-H). Although there were not any significant differences in this study, it is possible that differences exist given larger sample sizes or at the later, aged timepoint that was not available for this study in this region. A graphical depiction of these findings can be seen in Figure 5.

### Amygdala – mitochondrial isolation

**Figure 6:**
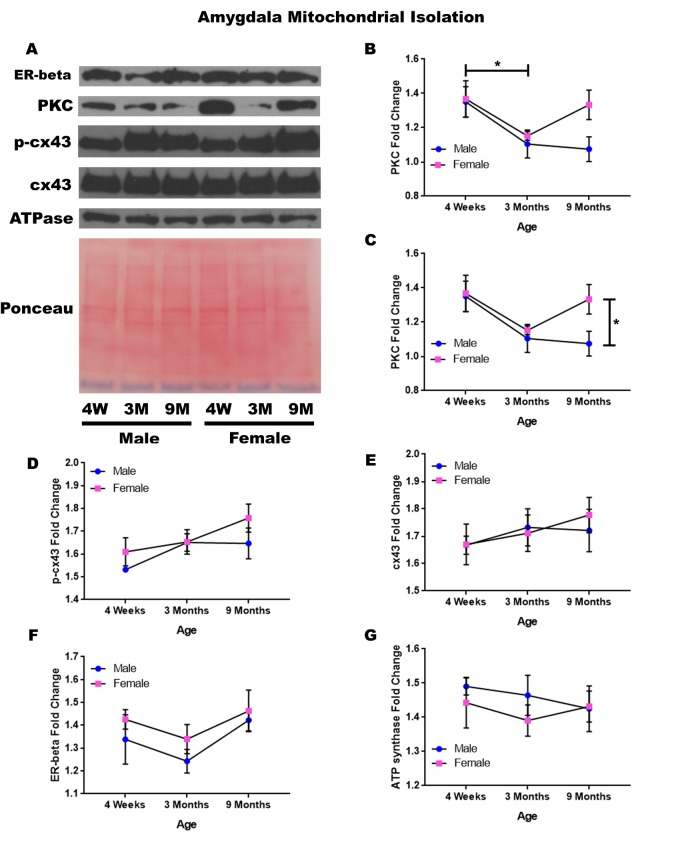
**Signaling downstream of estrogen in the mitochondria of the amygdala across age and gender**. Representative western blots for each protein of interest and a representative Ponceau stain as a load control (**A**). Graphical depiction of the fold change for PKC comparing age (**B**), PKC comparing gender (**C**), p-cx43 (**D**), cx43 (**E**), estrogen receptor beta (**F**), ATP synthase (**G**). Error bars = SEM. 4W = 4 weeks of age; 3M = 3 months of age; 9M = 9 months of age; 12M = 12 months of age. Pink = female; blue = male. ANOVA with Tukey posthoc, * = P<0.05.

In order to determine whether signaling downstream of the estrogen receptor changed within the mitochondria from the amygdala over time and between genders, mitochondria were isolated from the amygdala of male and female rats at 3 timepoints throughout their lifespan (4 weeks, 3 months, and 9 months of age; n = 3/gender/age). Although whole cell analysis for the amygdala did not show any significance, mitochondrial isolation for the amygdala demonstrates age-dependent and gender-dependent alterations. There was a significant decrease in the concentration of PKC between four weeks of age and three months of age (Figure 6A & 6B; p = 0.036). Additionally, 9-month female rats had significantly higher mitochondrial PKC when compared to their male counterparts (Figure 6A & 6C; p = 0.042). A trend for decreasing PKC in male rats from four weeks of age to nine months of age was also noted (Figure 6A & 6C; p = 0.077).

A trend for increased mitochondrial p-cx43 between four weeks of age to nine months of age was also found (Figure 6A & 6D; p = 0.067). Besides these significant differences and observed trends, it was noted that the concentration of mitochondrial cx43 and estrogen receptor beta did not significantly change in the amygdala and did not seem to be age-dependent or gender-dependent (Figure 6A, 6B, 6F). All of this data can be visualized in Figure 6.

### Corpus callosum – whole cell

**Figure 7:**
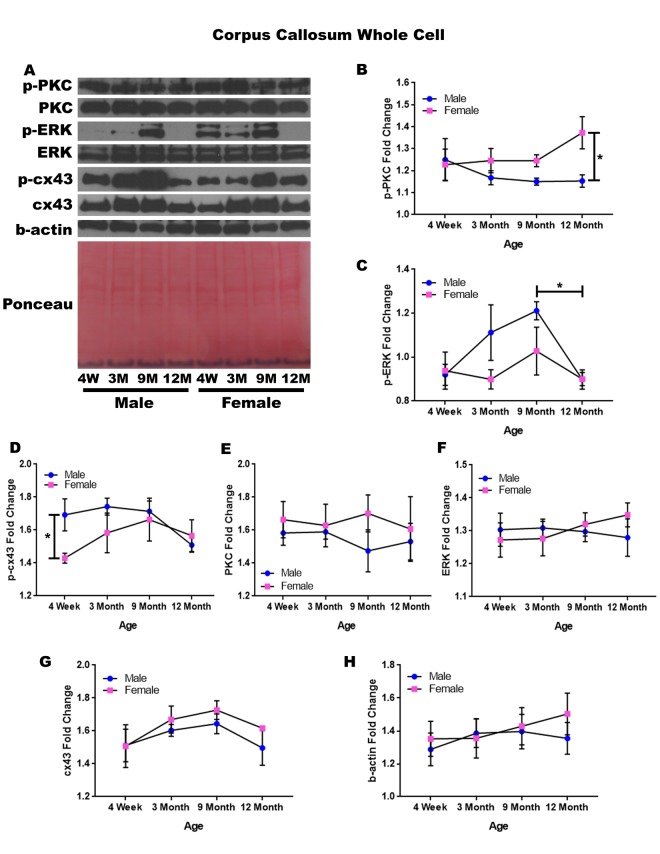
**Signaling downstream of estrogen in the corpus callosum across age and gender**. Representative western blots for each protein of interest and a representative Ponceau stain as a load control (**A**). Graphical depiction of the fold change for p-PKC (**B**), p-ERK (**C**), p-cx43 comparing gender (**D**), PKC (**E**), ERK (**F**), cx43 (**G**), beta-actin (**H**). Error bars = SEM. 4W = 4 weeks of age; 3M = 3 months of age; 9M = 9 months of age; 12M = 12 months of age. Pink = female; blue = male. ANOVA with Tukey posthoc, * = P<0.05.

In order to determine whether signaling downstream of the estrogen receptor changed within the corpus callosum over time and between genders, whole cell lysate proteins were isolated from the corpus callosum of male and female rats at 4 timepoints throughout their lifespan (4 weeks, 3 months, 9 months, and 12 months of age; n = 3/gender/age). The corpus callosum was yet another brain region that demonstrated significant age-dependent and gender-dependent changes within the whole cell analysis. Female rats at twelve months of age had significantly higher p-PKC than their male counterparts (Figure 7A & 7B; p = 0.014). There was also a significance decrease in the concentration of p-ERK between nine months of age and twelve months of age (Figure 7A & 7C; males, p = 0.040).

When comparing genders, male rats experienced notably higher concentrations of p-cx43 compared to the female rats at four weeks of age (Figure 7A & 7D; p = 0.047). Although statistical significance was not demonstrated, the data indicated that gender differences may exist within p-ERK at three months of age and nine months of age given a larger sample size (Figure 7A & 7C; p =0.058 and p = 0.098, respectively). This may also hold true for male rats between four weeks of age and nine months of age, as the concentration of p-ERK increased within this time period (Figure 7A & 7C; p = 0.057).

No significant differences were noted for PKC, ERK, and cx43 when comparing gender and age (Figure 7A, 7E, 7F, & 7G). A graphical depiction of this data is presented in Figure 7.

### Corpus callosum - mitochondrial isolation

**Figure 8:**
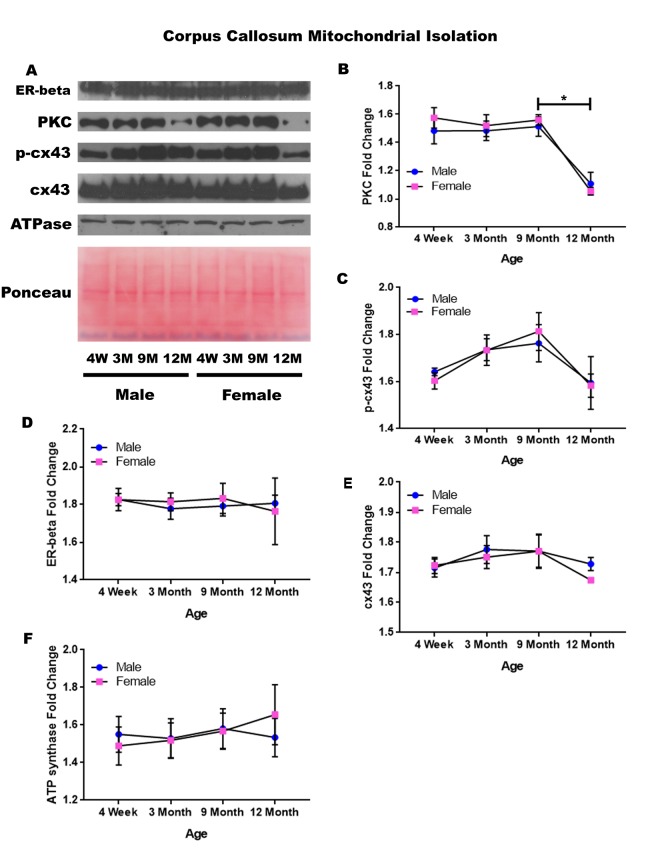
**Signaling downstream of estrogen in the mitochondria of the corpus callosum across age and gender**. Representative western blots for each protein of interest and a representative Ponceau stain as a load control (**A**). Graphical depiction of the fold change for PKC (**B**), p-cx43 (**C**), estrogen receptor beta (**D**), cx43 (**E**), ATP synthase (**F**). Error bars = SEM. 4W = 4 weeks of age; 3M = 3 months of age; 9M = 9 months of age; 12M = 12 months of age. Pink = female; blue = male. ANOVA with Tukey posthoc, * = P<0.05.

In order to determine whether signaling downstream of the estrogen receptor changed within corpus callosum mitochondria over time and between genders, mitochondria were isolated from the corpus callosum of male and female rats at 4 timepoints throughout their lifespan (4 weeks, 3 months, 9 months, and 12 months of age; n = 3/gender/age). Unlike the whole cell analysis, the mitochondrial isolation analysis for the corpus callosum only demonstrated age-dependent changes. No significant gender differences were found. PKC significantly decreased in both genders between nine months of age and twelve months of age (Figure 8A & 8B; male, p = 0.005; female, p = 0.002). A similar trend was noted in the concentration of p-cx43, which also seemed to be decreased between nine months of age and twelve months of age (Figure 8A & 8C; p = 0.059), although not statistically significant in this study. The concentration of estrogen receptor beta and cx43 did not significantly vary throughout the aging process or across gender lines (Figure 8A, 8D, & 8E). A graphical depiction of these findings can be visualized in Figure 8.

### Cerebellum – whole cell

**Figure 9:**
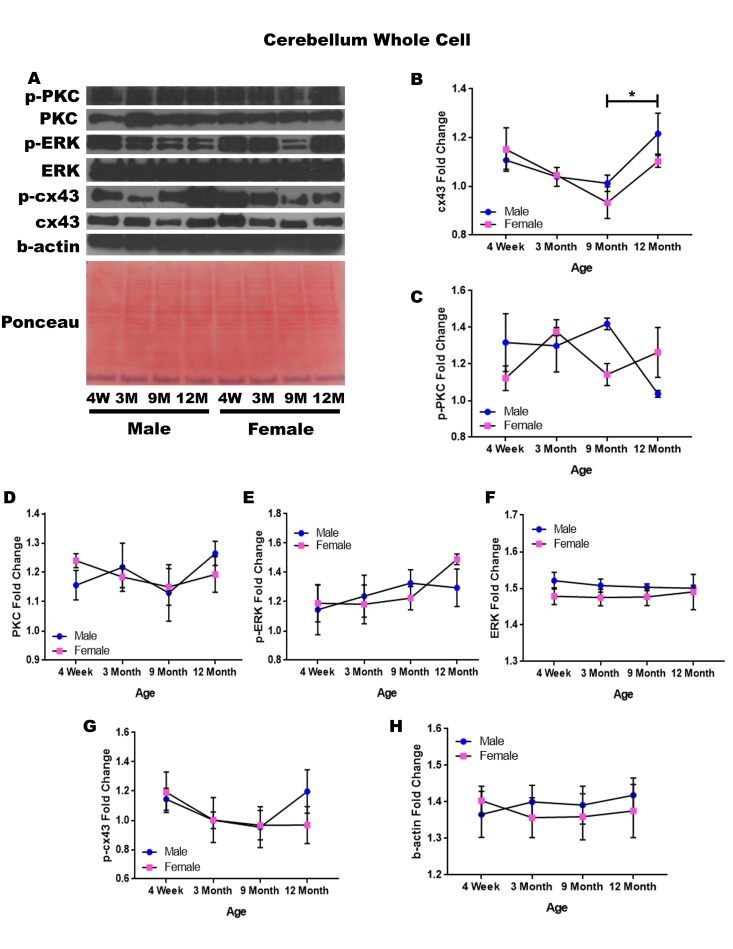
**Signaling downstream of estrogen in the cerebellum across age and gender**. Representative western blots for each protein of interest and a representative Ponceau stain as a load control (**A**). Graphical depiction of the fold change for for cx43 with age (**B**), p-PKC (**C**), PKC (**D**), ERK (**E**),ERK (**F**), p-cx43 (**G**), beta-actin (**H**). Error bars = SEM. 4W = 4 weeks of age; 3M = 3 months of age; 9M = 9 months of age; 12M = 12 months of age. Pink = female; blue = male. ANOVA with Tukey posthoc, * = P<0.05.

In order to determine whether signaling downstream of the estrogen receptor changed within the cerebellum over time and between genders, whole cell lysate proteins were isolated from the cerebellum of male and female rats at 4 timepoints throughout their lifespan (4 weeks, 3 months, 9 months, and 12 months of age; n = 3/gender/age). Cerebellar whole cell analysis illuminated that the concentration of cx43 is age-dependent in this region of the brain. It has been commonly known that cx43 decreases with age; therefore, the decrease in cx43 in both genders between four weeks of age and nine months of age, although not statistically significant, was not surprising. However, it was surprising to find that within the cerebellum cx43 significantly increased between nine and twelve months of age (Figure 9A & 9B; p = 0.017).

While the concentration of p-PKC did not show significant differences, it did reveal the possibility of gender differences at nine months of age, where males experienced increased levels of this protein (Figure 9A & 9C; p = 0.058). There is also the possibility that males have decreased p-PKC between nine months of age and twelve months of age, although again the threshold for statistical significance was not obtained (Figure 9A & 9C; p = 0.054). No significant differences were noted in cerebellar PKC, p-ERK, ERK, and p-cx43 regardless of age or gender (Figure 9A, 9C, 9D, 9E, & 9G). Data and controls are depicted graphically in Figure 9.

### Cerebellum – mitochondrial isolation

**Figure 10:**
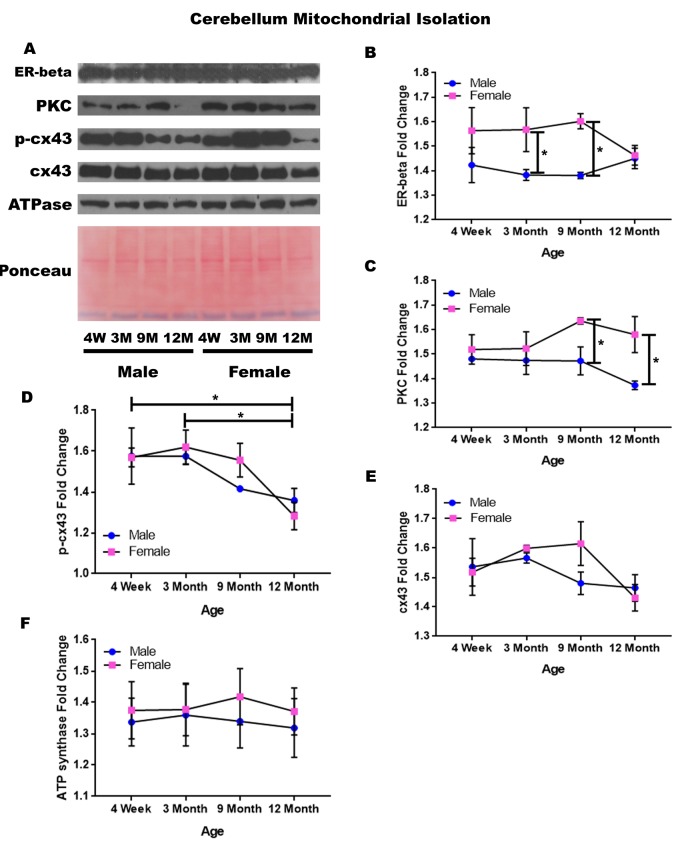
**Signaling downstream of estrogen in the mitochondria of the cerebellum across age and gender**. Representative western blots for each protein of interest and a representative Ponceau stain as a load control (**A**). Graphical depiction of the fold change for estrogen receptor beta (**B**), PKC (**C**), p-cx43 (**D**), cx43 (**E**), ATP synthase (**F**). Error bars = SEM. 4W = 4 weeks of age; 3M = 3 months of age; 9M = 9 months of age; 12M = 12 months of age. Pink = female; blue = male. ANOVA with Tukey posthoc, * = P<0.05.

In order to determine whether signaling downstream of the estrogen receptor changed within cerebellum mitochondria over time and between genders, mitochondria were isolated from the cerebellum of male and female rats at 4 timepoints throughout their lifespan (4 weeks, 3 months, 9 months, and 12 months of age; n = 3/gender/age). Contrary to the cerebellar whole cell analysis, significant age-dependent and gender-dependent alterations were found in the mitochondria of the cerebellum. Females had significantly higher mitochondrial estrogen receptor beta when compared to age-matched males at three months of age (Figure 10A & 10B; p = 0.041) and at nine months of age (Figure 10A & 10B; p = 0.018). Similarly, females had significantly higher PKC when compared to age-matched males at nine months of age (Figure 10A & 10C; p = 0.030) and at twelve months of age (Figure 10A & 10C; p = 0.015).

Beyond the gender-related changes, age-related changes were seen within the concentration of p-cx43. P-cx43 was significantly decreased between four weeks and twelve months of age (Figure 10A & 10D; p = 0.028), as well as between three and twelve months of age (Figure 10A & 10D; p = 0.015). In the cerebellum, the only protein investigated that did not significantly vary throughout the aging process or between genders was cx43 (Figure 10A & 10E). A graphical depiction of these findings can be visualized in Figure 10.

### Summary

A summary table indicating significant differences and trends for each brain region and signaling protein data can be found in Tables 1-4. Table 1 displays whole cell lysate protein concentration differences between males and females for each brain region. Table 2 displays significant differences in mitochondrial protein concentration differences between males and females for each brain region. Table 3 summarizes significant changes with age in the whole cell lysate protein concentrations for each brain region regardless of gender. Table 4 summarizes significant changes with age in the mitochondrial proteins for each brain region regardless of gender. Statistically significant differences, p<0.05, are indicated in yellow. Non-significant trends with a p value between 0.05 and 0.10, are indicated in light green.

**Table 1:** Summary of significant whole cell protein changes with gender.

	**Cortex**	**Hippocampus**	**Corpus Callosum**	**Cerebellum**
**PKC**		**4wk M>F**		
		**9mo M>F**		
		**12mo M>F**		
**p-PKC**		**4wk M>F**	**12mo F>M**	**9mo M>F**
		**12mo F>M**		
**ERK**				
**p-ERK**	**9mo M>F**		**3mo M>F**	
	**12mo M>F**		**9mo M>F**	
**cx43**		**12mo F>M**		
**p-cx43**			**4wk M>F**	

n = 3-4/age/gender/group. Yellow = p<0.05. Green = trend but non-significant

**Table 2:** Summary of significant mitochondrial protein changes with gender.

	**Cortex**	**Hippocampus**	**Amygdala**	**Corpus Callosum**	**Cerebellum**
**ER-beta**	**4wk F>M**				**3mo F>M**

**9mo F>M**
**PKC**			**9mo F>M**		**9mo F>M**
					**12mo F>M**
**cx43**		**9mo F>M**			
**p-cx43**	**12mo M>F**	**9mo F>M**			

n = 3-4/age/gender/group. Yellow = p<0.05. Green = trend but non-significant

**Table 3:** Summary of significant whole cell protein changes with Age regardless of gender.

	**Cortex**	**Hippocampus**	**Corpus Callosum**	**Cerebellum**
**PKC**		**Inc 4wk-3mo**		
**p-PKC**		**Dec 9mo-12mo**		**Dec 9mo-12mo**
**ERK**				
**p-ERK**			**Inc 4wk-9mo**	
			**Dec 9mo-12mo**	
**cx43**		**Inc 4wk-12mo**		**Dec 4wk-9mo**
				**Inc 9mo-12mo**
**p-cx43**				

n = 3-4/age/gender/group. Yellow = p<0.05. Green = trend but non-significant

**Table 4:** Summary of significant mitochondrial protein changes with age regardless of gender.

	**Cortex**	**Hippocampus**	**Amygala**	**Corpus Callosum**	**Cerebellum**
**ER-beta**		**Dec 9mo-12mo**			
**cx43**	**Inc 4wk-9mo**	**Inc 4wk-3mo**			
		**Dec 9mo-12mo F**			
**p-cx43**	**Inc 4wk-3mo**	**Inc 4wk-3mo**	**Inc 4wk-9mo**	**Dec 9mo-12mo**	**Dec 4wk-12mo**
		**Dec 9mo-12mo**			**Dec 3mo-12mo**
**PKC**			**Dec 4wk-3mo**	**Dec 9mo-12mo**	
			**Dec 4wk-9mo**		

n = 3-4/age/gender/group. Yellow = p<0.05. Green = trend but non-significant

## DISCUSSION

Females have a longer life expectancy than males in mammals, including humans [29,30]. Because this prolonged lifespan is not exclusive to humans, socioeconomic factors cannot be entirely to blame. This hints that a gender-specific biological differences, such as hormone regulation, may contribute to this phenomenon. Similarly, epidemiological studies have shown that there are gender- and age-dependent factors that correspond to the incidence and/or severity of various age-related neurodegenerative diseases. For example, males have an earlier onset and increased prevalence for Parkinson’s disease (PD) than age-matched females [31]. Likewise, females have been shown to have an increase in neuroprotection from stroke severity pre-menopause [7–9]. These gender-related studies have led to the hypothesis that the positive effects of estrogen on cellular processes may be one mechanism by which females have early life protection from neurodegenerative symptoms, and ultimately a longer lifespan. The mechanism(s) by which estrogen is neuroprotective is unknown; however, it has been well documented that estrogen can modify immune competence, reactive oxygen species (ROS) production, and telomere maintenance [32,33].

The loss of estrogen midlife, post-estrus, may argue that this neuroprotection declines at menopause. In fact, evidence does show that post-menopausal women have a higher incidence of Alzheimer’s Disease (AD) than men, as well as a faster cognition decline after disease onset [34,35]. Furthermore, the severity of stroke increases dramatically post menopause [7–9]. In addition, Bake S & Sohrabji F. showed that the increased Blood Brain Barrier permeability in aged, post-estrus rats could not be rescued with estrogen replacement therapy as it could in young females post ovarectomy [36] – suggesting that there are alterations post-estrus that do not allow the cells to respond to estrogen replacement therapy as in younger animals. These data suggest that loss of estrogen can indeed decrease neuroprotection in females. Since females tend to live longer, this may seem counter-intuitive. However, longer lifespan has also been correlated to later stage menopause [37], which may suggest that the lifetime cumulative exposure to estrogen exerts a long-lasting effect. Altogether, we suggest that gender and hormone regulation are biological variables that may be important when considering both normal aging and age-associated neurodegenerative diseases.

The mechanism(s) for hormone-associated neuroprotection and the dramatic decline in females at menopause is yet unknown. The current study examined cell signaling molecules downstream of the estrogen receptor-beta over the lifespan of both male and female rats to attempt to elucidate significant changes that occur during the post-estrus change.

We propose that gender-dependent hormone fluctuations with age may be one factor that determines why neuroprotection differs between males and females, which can be more pronounced during the non-estrus, or menopausal stage of female life. The decline in estradiol noted in the aged (9-month and 12-month female rats) corresponded with significant changes in PKC, p-PKC, p-ERK and cx43 protein concentrations throughout the brain, but most notably within the hippocampus of female rats during those timeframes. Decreased expression of PKC isoforms with age in the hippocampus and frontal cortex have been linked to significant declines in spatial memory [6]. Furthermore, in a 2016 review Orellana JA et al. expound upon the necessity of cx43 for memory consolidation throughout the brain [38]. However, these previous studies did not differentiate between gender. Our findings show significant gender-dependent alterations within both PKC and cx43 within the hippocampus and cortex. Moreover, given the historical links of PKC and cx43 to memory during neurodegeneration and aging, the correlations provided from this study indicate the possibility of linking these pathway changes to the memory alterations noted and further explain the gender-dependent changes at this age in females. Further experiments to define whether a decline in memory is hormone-dependent in females at this time are planned.

Data from this study also correlates the decrease in estradiol levels with significant differences between male and female cx43 and p-cx43 in hippocampal mitochondria, and mitochondrial PKC concentrations within the cerebellum. Mitochondrial PKC and cx43 have been previously shown to regulate oxidative phosphorylation in the mitochondria [39–41]; therefore, an age-dependent decline in mitochondrial cx43 and p-cx43 would be indicative of non-optimal mitochondrial respiration often found in neurodegenerative diseases. This is consistent with previous reports that showed attenuation of oxidative stress and mitochondrial dysfunction were sufficient to improve the memory in a mouse model for Alzheimer’s Disease [42]; and suggests that the loss of hormones at this critical time of life may contribute to the increased cognitive and learning and memory deficit severity noted in this population due to mitochondrial signaling alterations.

Mitochondrial cx43 and p-cx43 significantly increased as the rats aged between the adolescent 4-week age and more adult 3-month to 12-month ages in the cortex and hippocampus regardless of gender. Significant decreases in these protein concentrations were not found until the 9 and 12-month post-estrus age in female rats, and this decrease was gender-specific. It is interesting to note here that these alterations in cx43 and p-cx43, which both correspond to the ages of neuroprotection and memory development early in life and decline with age, are only found in the mitochondrial fractions and were not found to be significantly different in the whole cell lysates. Similarly, PKC from the mitochondrial isolates but not whole cell lysates also significantly decreased in the corpus callosum at the 9 and 12-month, aged timepoint regardless of gender. These discoveries are important because while PKC and cx43 have been associated with age and memory loss, the distinction between the mitochondrial function and that of signaling elsewhere in the cell is novel.

PKC and cx43 are both known down-stream targets of estrogen and are known to regulate mitochondrial respiration. These proteins experienced expression changes throughout the aging process, some of which were gender-dependent. These expression changes varied based on the localization of the protein, within whole cell lysate or isolated mitochondria, suggesting that the altered protein concentration is likely secondary to epigenetic changes rather than genetic changes. Finding statistical significance with such a small sample size using an in vivo rat model elucidates just how dramatic some of these alterations are.

**Figure 11:**
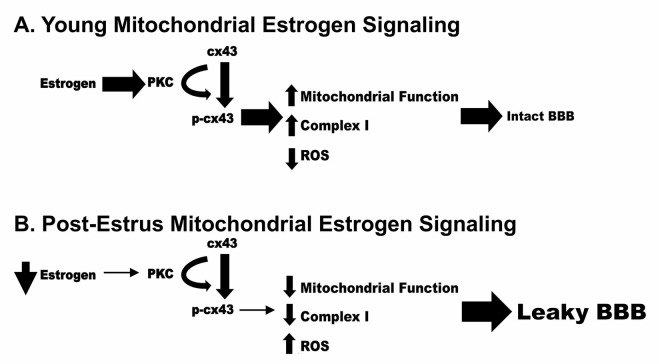
Schematic representation of a potential mechanism by which loss of estrogen post estrus can result in decreased mitochondrial function and increased permeability of the Blood Brain Barrier.

While the current study does not focus upon the Blood Brain Barrier, disruption of this barrier has been correlated to normal aging as well as many neurodegenerative diseases. Mitochondrial dysfunction has also been highly correlated to neurodegenerative disease and Blood Brain Barrier disruption. Therefore, we propose a potential mechanism by which loss of estrogen post estrus can result in decreased mitochondrial function and increased permeability of the Blood Brain Barrier (Figure 11). In this model, loss of estrogen post menopause results in a reduction of pPKC and p-cx43 leading to decreased mitochondrial function, increased ROS production and ultimately an increase in Blood Brain Barrier permeability.

Not only do each of the brain regions investigated demonstrate unique age-dependent alterations, but they also all demonstrate gender-dependent alterations. Although numerous cellular signaling pathways may be involved in these alterations, signaling downstream of the estrogen receptor-beta seems to have a pronounced role. Several of the proteins involved within this signaling cascade experienced expression changes throughout the aging process. Of the proteins examined, only ERK remained relatively constant throughout all age groups, genders, and brain regions.

Data from this study also correlates the decrease in estradiol levels with significant differences between male and female cx43 and p-cx43 in hippocampal mitochondria, and mitochondrial PKC concentrations within the cerebellum. This fits with previous reports where attenuation of oxidative stress and mitochondrial dysfunction were sufficient to improve the memory in a mouse model for Alzheimer’s Disease [42]; and suggests that the loss of hormone at this critical time of life may contribute to the increased cognitive and learning and memory deficit severity noted in this population due to mitochondrial signaling alterations.

Aging is an extremely intricate, complex process that involves changes in a variety of genes, protein signaling pathways, and epigenetic factors. It is noted that we only examined a small subset of estrogen-responsive proteins; however, each brain region studied did demonstrated unique age-dependent and gender-dependent signaling protein concentration alterations. These significant protein concentration changes will allow for focused region-specific future studies that will help unravel mechanisms behind neuroprotection and memory changes with age and gender.

## MATERIALS AND METHODS

### Animals and tissue harvesting

Forty-seven Sprague Dawley rats were obtained (Charles River Laboratories) and were split into four age groups (4 weeks, 3 months, 9 months, and 12 months of age), for both males and females. Each of these groupings were repeated so that tissue could be isolated for both total cellular analysis and for mitochondrial fractionalization studied (n=3-4 per group).

Blood was collected from each animal after euthanasia but before cardio-perfusion via a cardiac draw. Elisa assays for estradiol levels were performed to correlate the age-associated decline in estradiol levels and to assess post-estrus status in 9-12 month old females (data not shown). Each rat was euthanized using an intraperitoneal injection of phenobarbital. A foot pinch or tail pinch test was performed to ensure no withdrawal reflex was elicited, and to confirm that the rat had been appropriately anesthetized. Cardio-perfusion was then performed using ice cold 1× phosphate buffered saline (PBS). Brain regions were dissected, flash frozen in liquid nitrogen, and stored at -80°C.

### Tissue homogenization - whole cell

Tissue samples were homogenized in lysis buffer containing 1% phosphatase inhibitor cocktail 2, 1% phosphatase inhibitor cocktail 3, 1% phenylmethylsulfonyl fluoride, and 4% cOmplete EDTA-free protease inhibitor cocktail. They were then centrifuged for ten minutes (12,000 rpm at 4°C), and the pellet and supernatant were frozen and stored at -80°C.

### Mitochondrial isolation

This mitochondrial isolation procedure is a modified version of the mitochondrial isolation protocol provided by Cold Springs Harbor Laboratory Press (Cold Spring Harb Protoc; doi:10.1101/pdb.prot080002). Samples were incubated in RBS hypobuffer containing 1% phosphatase inhibitor cocktail 2, 1% phosphatase inhibitor cocktail 3, 1% phenylmethylsulfonyl fluoride , and 4% cOmplete EDTA-free protease inhibitor cocktail for five minutes before being homogenized for ten minutes using a micro tube homogenizer. Immediately after homogenization, 2.5×MS homogenization buffer was added to each sample. The samples were mixed by inversion five times, transferred to a 1.5 mL microcentrifuge tube, centrifuged for five minutes (1,300 g at 4°C), and the supernatant was transferred to a new 1.5 mL microcentrifuge tube. This process was performed three times, transferring the supernatant to a new 1.5 mL microcentrifuge tube each time. After the third round of centrifugation supernatant was then centrifuged for fifteen minutes (12,000 g at 4°C). The remaining supernatant after this final centrifugation was transferred to a new 1.5 mL microcentrifuge tube. The pellet from the final centrifugation was resuspended with 100 µL of 1×MS homogenization buffer (1% phosphatase inhibitor cocktail 2, 1% phosphatase inhibitor cocktail 3, 1% phenylmethylsulfonyl fluoride, and 4% cOmplete EDTA-free protease inhibitor cocktail). The three pellets, the final supernatant, and the resuspended sample were all frozen and stored at -80°C.

### Western blotting

A BCA protein assay was performed to determine the total protein concentration of each sample using a Pierce BCA Assay Protein Kit. Western blots were performed at described previously [43]. The primary antibodies that were used throughout this study were: 1:1,000 Anti-Phospho-PKC alpha (Ser657) goat polyclonal (Santa Cruz Biotechnologies, Product number: sc-12356), 1:1,000 Anti-PKC alpha rabbit polyclonal (Cell Signaling Technology, Product number: 2056S), 1:1,000 Anti-Phospho-p44/42 ERK (Thr202/Tyr204) XP rabbit monoclonal (Cell Signaling Technology, Product number: 4370S), 1:1,000 Anti-p44/42 ERK (L34F12) mouse monoclonal (Cell Signaling Technology: Product number: 4696S), 1:500 Anti-Estrogen receptor beta rabbit polyclonal (Invitrogen, Product number: PA1-310B), 1:1,000 Anti-Phospho-Connexin 43 (Ser368)(D6W8P) rabbit monoclonal (Cell Signaling Technology, Product number: 52559S), 1:8,000 Anti-Connexin 43 (GJA1) rabbit polyclonal (Abcam, Product number: ab11370), 1:1,000 Anti-ATP5B (R-20) goal polyclonal (Santa Cruz Biotechnology, Product number: SC-16689), and 1:5,000 Anti-Beta-actin (13E5) rabbit monoclonal (Cell Signaling Technology, Product number: 4970S). The secondary antibodies that were used throughout this study were: 1:4,000 Donkey Anti-Goat Horse Radish Peroxidase (HRP) conjugated IgG (Santa Cruz Biotechnologies, Product number: sc-2033), 1:5,000 Anti-Mouse HRP-conjugated IgG (Cell Signaling Technology, Product number: 7076S), and 1:5,000 or 1:10,000 Anti-Rabbit HRP-conjugated IgG (Cell Signaling Technology, Product number: 7074S).

Each membrane utilized two processes to verify even protein loading to each well. All blots were stained with Ponceau as a total protein loading control. For mitochondrial isolation, membranes were also probed with 1:1,000 Anti-ATP5B antibody. For whole cell lysates, the load controls utilized beta-actin (1:5000).

### Analysis

X-ray film was scanned using a HP Scanjet 3970, grayscale coloring, and a resolution of 1200 dpi. Bands were quantified using ImageJ Software, and band intensities were normalized as a ratio to corresponding load control values. A two-way ANOVA with Tukey Post-hoc testing was performed to determine statistical significance using SigmaPlot v14.0. Line graphs were created using GraphPad Prism 7 and demonstrate the mean ± standard error of mean for each group.

## Supplementary Material

Supplementary Table
