# Supplementary material for: Gender- and region-specific changes in estrogen signaling in aging rat brain mitochondria
Source: Aging (Albany NY). 2018 Aug 31;10(8):2148–69. doi: 10.18632/aging.101538 (PMC6128413; doi:10.18632/aging.101538)
Supplement: Supplementary Table [file aging-10-101538-s001.pdf]

## SUPPLEMENTARY TABLE

**Supplementary Table 1.**

| Aging Stage | Rat Age | Human Age |
|---|---|---|
| Adolescent | 0-6 weeks | 0-12 years |
| Puberty | 5-8 weeks | 12-18 years |
| Young Adult | 8 weeks – 11 months | 18-40 years |
| Middle-Aged Adult | 11-15 months | 50-60 years |
| Elderly | Past 15 months | Past 60 years |
